# Errata

**DOI:** 10.1200/JCO.2016.71.6175

**Published:** 2017-01-10

The July 10, 2016, article by Denduluri, et al, entitled “Selection of Optimal Adjuvant Chemotherapy Regimens for Human Epidermal Growth Factor Receptor 2 (HER2) -Negative and Adjuvant Targeted Therapy for HER2-Positive Breast Cancers: An American Society of Clinical Oncology Guideline Adaptation of the Cancer Care Ontario Clinical Practice Guideline” (J Clin Oncol 34: 2416-2427, 2016), contained an error.

Under Recommendations in the Bottom Line section, based on the findings of the trial conducted by the Gruppo Italiano Mammella investigators, which demonstrated improved disease-free survival and overall survival observed with four cycles of epirubicin 90 mg/m2 and cyclophosphamide 600 mg/m2 followed by four cycles of paclitaxel 175 mg/m2 delivered every two weeks (dose-dense EC-P) compared to every 3 weeks, the guideline adaptation should have included the dose-dense EC-P regimen, as provided below, in the list of acceptable adjuvant chemotherapy regimens for patients with higher-risk early breast cancer.

**Dose-dense epirubicin 90mg/m2, cyclophosphamide 600mg/m2 every 2 weeks 4 cycles → paclitaxel 175mg/m2 every 2 weeks for 4 cycles.**

The online version has been corrected in departure from the print. The authors regret the omission.

